# Gas Void Fraction Measurement of Gas-Liquid Two-Phase CO_2_ Flow Using Laser Attenuation Technique

**DOI:** 10.3390/s19143178

**Published:** 2019-07-19

**Authors:** Haochi Wu, Quansheng Duan

**Affiliations:** School of Control and Computer Engineering, North China Electric Power University, Beijing 102206, China

**Keywords:** carbon capture and storage, gas-liquid two-phase CO_2_ flow, void fraction, laser attenuation, photodiode sensor array

## Abstract

The carbon capture and storage (CCS) system has the potential to reduce CO_2_ emissions from traditional energy industries. In order to monitor and control the CCS process, it is essential to achieve an accurate measurement of the gas void fraction in a two-phase CO_2_ flow in transportation pipelines. This paper presents a novel instrumentation system based on the laser attenuation technique for the gas void fraction measurement of the two-phase CO_2_ flow. The system includes an infrared laser source and a photodiode sensor array. Experiments were conducted on the horizontal and vertical test sections. Two Coriolis mass flowmeters are respectively installed on the single-phase pipelines to obtain the reference gas void fraction. The experimental results obtained show that the proposed method is effective. In the horizontal test section, the relative errors of the stratified flow are within ±8.3%, while those of the bubble flow are within ±10.6%. In the vertical test section, the proposed method performs slightly less well, with relative errors under ±12.2%. The obtained results show that the measurement system is capable of providing an accurate measurement of the gas void fraction of the two-phase CO_2_ flow and a useful reference for other industrial applications.

## 1. Introduction

In recent decades, global warming and climate change have caused many extreme weather events, such as droughts, floods and melting glaciers due to excessive anthropogenic CO_2_ emissions [1,2]. The carbon capture and storage (CCS) system is proposed to reduce the emissions of CO_2_. CCS uses the carbon capture technique to separate CO_2_ from the emissions in industrial and energy-related processes and then stores the CO_2_ in the ocean, underground and other places [3]. The CCS technology is considered as one of the most important methods in dealing with the global climate change in the short term. Pipeline transportation is identified as the most economical and effective approach to transport the CO_2_ over long distances [4]. Unlike other fluids, the critical temperature of CO_2_ is very closer to the temperature of ambient environments. Therefore, a phase transition may occur due to the environment condition changes during transportation, which will result in the formation of a gas-liquid two-phase CO_2_ flow [5]. The gas void fraction is an important parameter of the gas-liquid two-phase CO_2_ flow. Therefore, a reliable and accurate measurement of the gas void fraction is necessary for the condition monitoring and operation optimization of the CCS process. However, a high pressure in transportation pipelines and complex flow conditions make the measurement more challenging.

A variety of typical methods, including capacitance sensors [6,7], wire-mesh sensors [8,9], radiation attenuation techniques [10,11], magnetic resonance [12,13] and ultrasonic techniques [14,15] have been applied to measure the gas void fraction of the gas-liquid two-phase flow. The sensing principle of the capacitance sensors makes use of different electrical permittivities of the phases. Such sensors have the advantages of a structural simplicity and low cost. Huang et al. [6] designed a dual needle-contact capacitance probe to measure the local void fractions and bubble velocities of the air-water two-phase flow. Li et al. [7] developed a method for the gas void fraction measurement and flow pattern recognition of the gas-oil two-phase flow based on a capacitance sensor and the least-squares support vector machine (LS-SVM) technique. However, the permittivity of gas and liquid CO_2_ is 1 and 1.6, respectively. The discrepancy between the gas and liquid phase CO_2_ is hard to distinguish. In recent years, wire-mesh sensors have been developed to investigate the gas void fraction using conductivity probes. Olerni et al. [8] applied a wire-mesh sensor and an electrical resistance tomography to measure the air distribution of an upwards air-water two-phase flow. Ofuchi et al. [9] presented the use of the wire-mesh sensor to study the flow behavior of the gas-oil two-phase flow. These intrusive sensors disturb the flow dynamics in pipelines. In recent studies, non-intrusive radiation attenuation and magnetic resonance techniques have been utilized to achieve the measurement of the gas-liquid two-phase flow. However, the disadvantages of high costs and unsafety restrict the applications. Ultrasonic methods are usually employed to measure the flow rate of the gas-liquid two-phase flow [14]. Addali et al. [15] proposed a method to measure the gas void fraction in the gas-liquid two-phase slug flow using the acoustic emission technology. The results have demonstrated that the information about the gas void fraction can be derived from the acoustic emission signals. Thus, ultrasonic methods are available for the gas void fraction measurement in two-phase CO_2_, but the high pressure in the pipeline may destroy the ultrasonic sensor.

Optical methods are superior to the above methods and are therefore attractive in achieving the gas void fraction measurement in CO_2_ pipelines due to the advantages of being non-intrusive, and having a rapid response and a high spatial resolution. Three conventional methods, based on the optical sensing techniques, are often adopted. They are the optical probe method, the visualization method and the laser-based method [16]. As an intrusive sensor, the optical probe [17] is directly in contact with the fluid and will therefore disturb the fluid. Moreover, the impurities in pipelines will contaminate the probe and increase the measurement error. The visualization method, based on high-speed cameras and tomography algorithms [18,19], has been well developed to measure the gas void fraction. However, the disadvantages of a high cost, complex construction and unsuitability in harsh environments restrict its applications. In recent studies [20,21], the laser-based method has been widely used to characterize the parameters of the gas-liquid two-phase flow. As a non-intrusive method, the laser-based method obtains the measurement results through detecting the changes of the optical properties of fluids. Duan et al. [22] proposed a laser-based method using an infrared ray to identify the flow patterns and detect the liquid slug in a horizontal gas-liquid pipe. The experimental results have demonstrated the effectiveness and accuracy of this technique. Mithran et al. [23] studied the effect of the behavior of infrared ray irradiation in the gas-liquid two-phase flow. Until now, the gas void fraction measurement of the gas-liquid two-phase flow using the laser-based method has been rarely reported.

In previous studies [24,25], it was found that the laser transmittance varies with the area of the gas-liquid surface when an infrared laser beam passes through the gas-liquid two-phase flow. Information about the gas void fraction of the two-phase flow can be derived from the laser transmittance. Therefore, this study aims to develop a method based on an infrared laser and detector array for the gas void fraction measurement of the two-phase CO_2_ flow.

## 2. Methodology

### 2.1. Sensor Design

**Figure 1:**
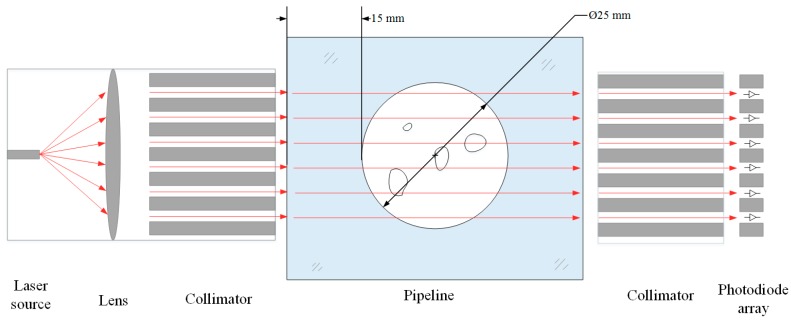
The structure of the sensing configuration.

Figure 1 shows the structure of the sensing configuration, which consists of a laser source, a convex lens, two collimators, a photodiode array sensor and a data acquisition unit. The laser source is placed at the focus of the convex lens to produce parallel incident beams. The incident beams are infrared light with a wavelength of 980 nm and an output power of 100 mW. The wavelength of the laser source is selected according to the penetrability of the laser diode. When the wavelength of the infrared ray is longer than 1064 nm, most of the light is reflected by the pipe wall, resulting in the inability to penetrate the pipe. The collimators ensure that the light passes through the pipe in parallel.

**Figure 2:**
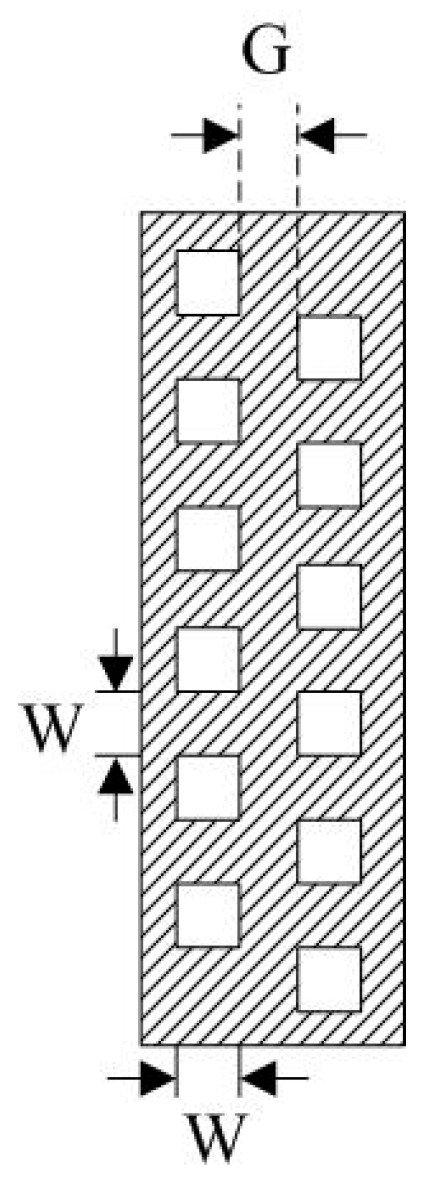
Layout of the collimator.

To avoid the dead space between the photodiodes, the collimators are designed with two layers, as shown in Figure 2. The two layers are parallel to each other and cross each other along the pipe axis in order to detect all the pipe cross-sections. After the parallel laser beams pass through the fluid, the intensities are reduced due to the absorption by the fluid. The collimators of the laser source and the detectors are arranged on the same axis so that the laser beams are guaranteed to be parallel and collimated. Each laser beam element has a width *W* of 8 mm and a uniform spacing *G* of 5 mm. The dead space between the photodiodes is avoided in the axial length range of *2W + G*.

### 2.2. Infrared Laser Measurement Principle

When passing through a uniform transparent medium, the parallel laser beam will attenuate. In a pipe filled with gas and liquid, the attenuation coefficients of gas and liquid have disparities for laser beams [26]. Based on the classic Lambert-Beer law, laser attenuation detection can be represented as

(1) $$I=I_{0}\times e^{-\mu d}$$

where *I_0_* and *I* are the intensities of the incident and transmitted laser beam, respectively. *μ* is the absorption coefficient, and *d* is the path length of the laser beam in the fluid.

When the parallel laser beam passes through the gas-liquid two-phase flow in the pipe, the normalized intensity of the laser beam *I_norm_* can be calculated from

(2) $$I_{norm}=\frac{I-I_{l}}{I_{g}-I_{l}}$$

where *I_l_* and *I_g_* are the intensities of the laser beams passing through the pure liquid and gas, respectively. The information of the gas void fraction is implicit in *I_norm_*.

When each parallel laser beam is well collimated and monochromatic, the relationship between the gas void fraction α and *I_norm_* can be expressed as

(3) $$\alpha =\frac{1}{N}\sum_{n=1}^{N}I_{norm}$$

where *N* is the number of the laser beams.

## 3. Experimental Section

**Figure 3:**
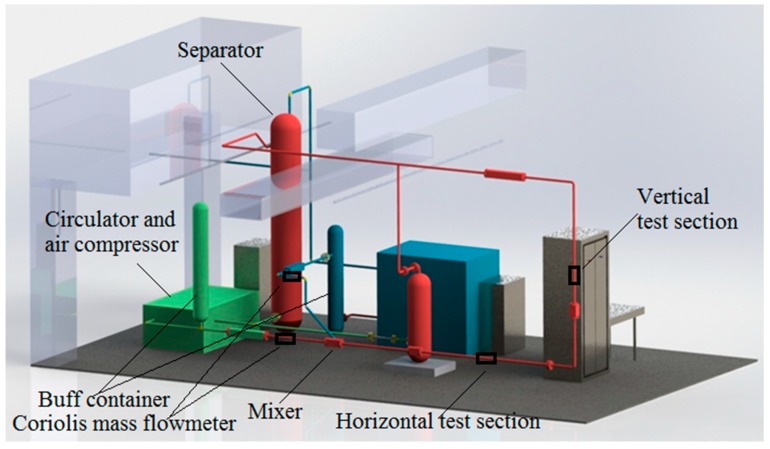
Schematic diagram of the gas-liquid two-phase CO_2_ flow test rig.

The experiments were conducted on the gas-liquid two-phase CO_2_ flow test rig. The structure of the test rig is shown in Figure 3. The gaseous and liquid CO_2_ fluids separately flow out from the upper and lower parts of the separator. The pump and air compressor separately drive the gaseous and liquid CO_2_ flow in the pipelines. The gaseous and liquid CO_2_ are fully mixed at the mixer to produce the gas-liquid two-phase CO_2_ flow with different void fractions. Then, the two-phase CO_2_ flow passes through the horizontal and vertical test sections and finally returns to the separator. Two sets of identical laser detection devices are respectively mounted on the horizontal and vertical test sections.

**Figure 4:**
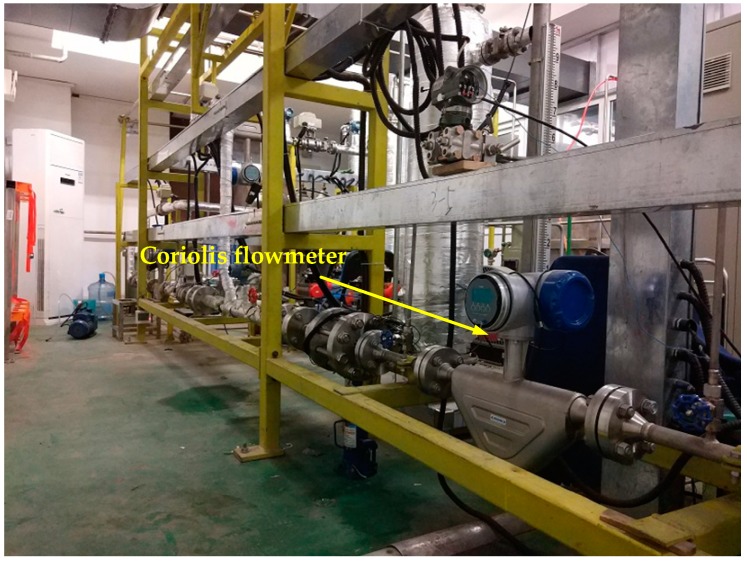
Installation of the Coriolis flowmeter on the test rig.

Two buffers are separately installed in the single-phase pipe sections to reduce the pulsation of the fluid and ensure that the experimental process is stable and continuous. For the purpose of reference, two Coriolis flowmeters, as shown in Figure 4, are respectively mounted downstream of the two buffers to measure the mass flowrate and the density of single gas and liquid phase CO_2_. Previous studies have shown that the Coriolis flowmeter can accurately measure the mass flowrate of single-phase CO_2_. The measurement uncertainties of the Coriolis flowmeters in the gas single-phase section and liquid single-phase section are 0.35% and 0.16%, respectively, which are accurate enough to provide the reference information. The temperature of the fluid in the pipeline ranges from 20~30 °C, and the pressure ranges from 5.7~7.2 MPa. The mass flowrate of liquid CO_2_ is set from 500 kg/h to 1300 kg/h, resulting in a reference gas void fraction ranging from 0% to 72%.

**Figure 5:**
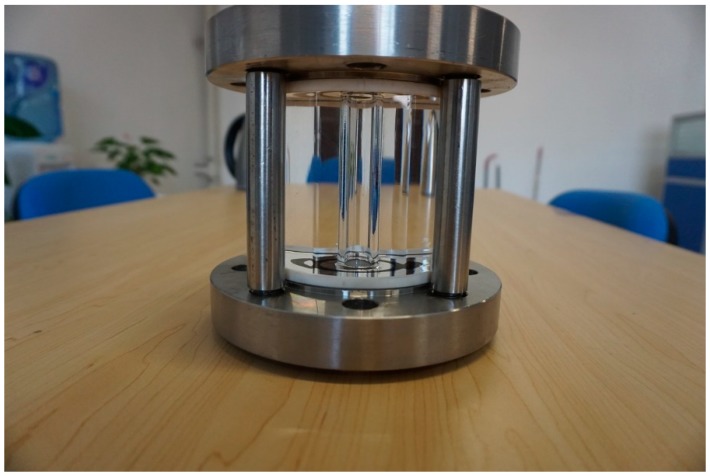
Photo of the observation window.

An observation window is installed downstream of the mixer. Figure 5 shows a photo of the observation window. It is made of polymethyl methacrylate (PMMA), with a high transmittance of laser beams. The observation window is designed to withstand the gas-liquid two-phase CO_2_ flow with an inner diameter of 25 mm and a wall thickness of 15 mm. The transmitted laser beam is captured by the photodiodes and converted into the voltage signals by the acquisition unit.

Ignoring the slip effect between the liquid and gas, the average gas void fraction is approximated to the gas volume fraction. Therefore, the average gas void fraction *α_r_* can be defined as

(4) $$\alpha_{r}=\frac{q_{mg}\rho_{l}}{q_{ml}\rho_{g}+q_{mg}\rho_{l}}$$

where *q_mg_* and *q_ml_* are the mass flowrates of the gas phase CO_2_ and liquid phase CO_2_, respectively, and *ρ_g_* and *ρ_l_* are the densities of the gas phase CO_2_ and liquid phase CO_2_, respectively. All these parameters are calculated from the measurements from the reference Coriolis mass flowmeters.

## 4. Experimental Results and Discussions

### 4.1. Flow Pattern Analysis

In order to obtain the normalized laser intensity *I_norm_*, the intensities of the laser beam passing through the pipe filled with pure gas and liquid, *I_g_* and *I_l_*, are measured. The experiments were simultaneously carried out on the horizontal and vertical test sections. Owing to the effect of gravity, the stratified and bubble flows are produced on the horizontal test section. The flow pattern in the vertical test section is mainly bubble flow. 

**Figure 6:**
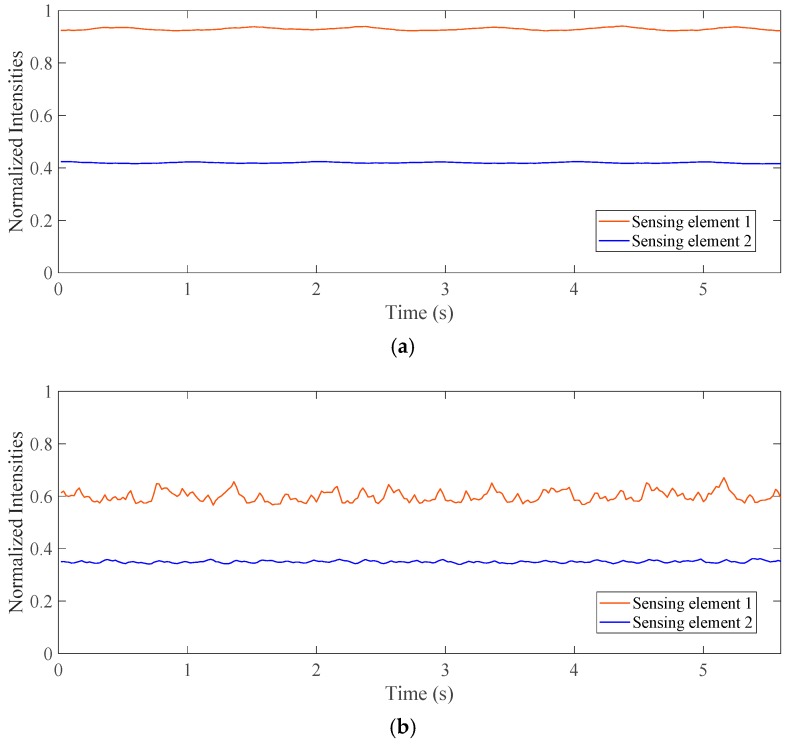

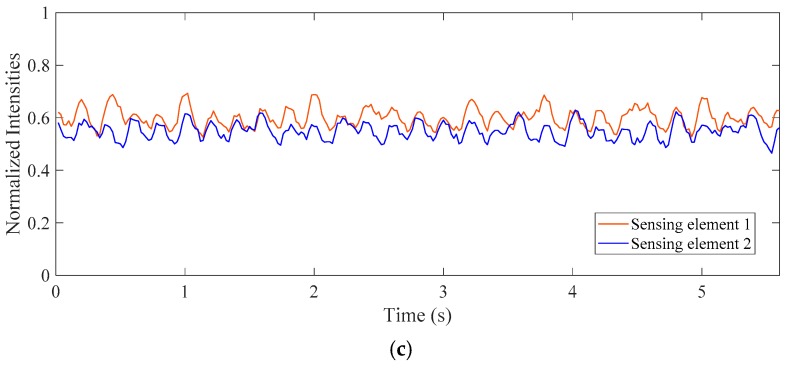
Normalized laser intensities of different patterns. (**a**) Stratified flow in the horizontal test section; (**b**) Bubble flow in the horizontal test section; (**c**) Bubble flow in the vertical test section.

The flow patterns of the gas-liquid two-phase CO_2_ flow have influences on the void fraction measurement. The effects of the flow patterns are investigated using two photodiode sensing elements, respectively placed in the top and bottom regions of the pipeline. Figure 6 depicts the normalized intensity of the laser beams of different patterns from two sensing elements. As can be seen from Figure 6a, the normalized intensities of the laser beam passing through the stratified flow remain stationary, but the decreased amplitudes are different. However, it is obvious that the normalized laser beam intensities passing through the bubble flow display fluctuations, as shown in Figure 6b,c. Because of the effect of gravity, the bubbles are mostly distributed in the upper part of the horizontal pipeline; thus, the normalized intensities of the laser beams passing through the top part of the pipeline have a stronger fluctuation than those passing through the bottom part. Moreover, both of the normalized intensities of the laser beams passing through the bubble flow in the vertical test section have a significant fluctuation; this is because the bubbles are evenly distributed in the vertical pipeline. Therefore, it is necessary to separately discuss the measurement accuracy for the different flow patterns.

### 4.2. Experimental Results in the Horizontal Test Section

**Figure 7:**
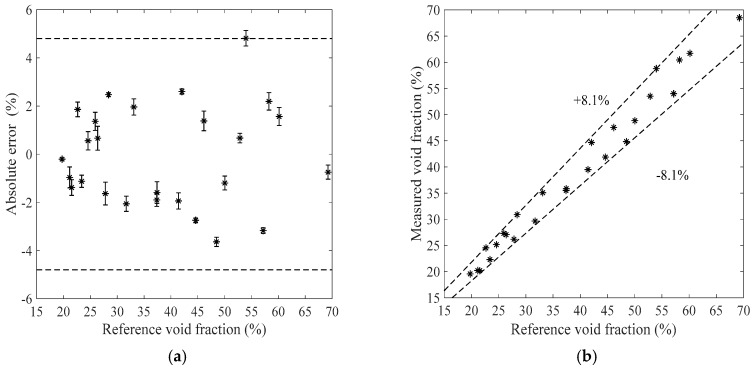
The measurement results of the stratified flow in the horizontal test section when the liquid mass flowrate is 500 kg/h. (**a**) Absolute errors of the void fraction; (**b**) Comparison between the measured and reference void fractions.

**Figure 8:**
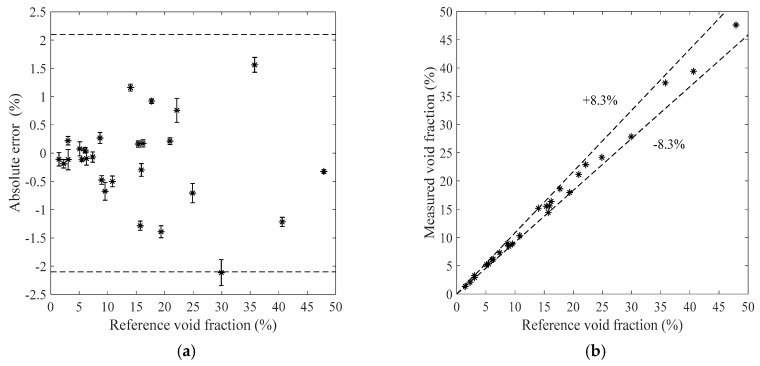
The measurement results of the stratified flow in the horizontal test section when the liquid mass flowrate is 900 kg/h. (**a**) Absolute errors of the void fraction; (**b**) Comparison between the measured and reference void fractions.

**Figure 9:**
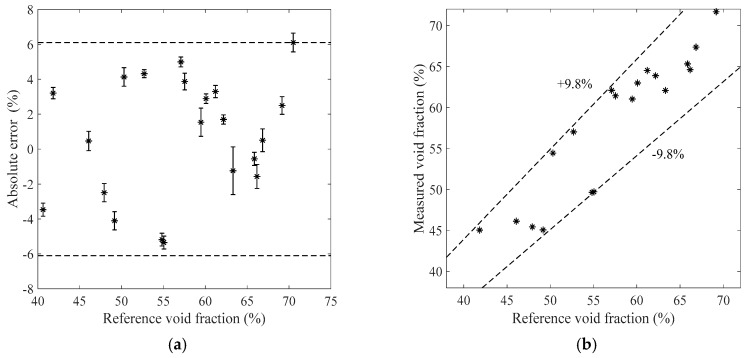
The measurement results of the bubble flow in the horizontal test section when the liquid mass flowrate is 1100 kg/h. (**a**) Absolute errors of the void fraction; (**b**) Comparison between the measured and reference void fractions.

**Figure 10:**
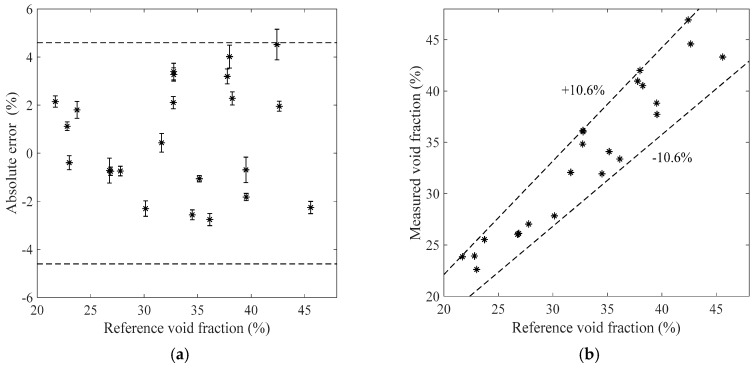
The measurement results of the bubble flow in the horizontal test section when the liquid mass flowrate is 1300 kg/h. (**a**) Absolute errors of the void fraction; (**b**) Comparison between the measured and reference void fractions.

Figure 7, Figure 8, Figure 9 and Figure 10 show the void fraction measurement results of the stratified flow and the bubble flow in the horizontal test section with various void fraction ranges. The maximum absolute error of the stratified flow is 4.8%, as shown in Figure 7a and Figure 8a, and its maximum relative error is ±8.3%, as shown in Figure 7b and Figure 8b. The maximum absolute error of the bubble flow is 6.2%, and its maximum relative error is ±10.6%, as illustrated in Figure 9 and Figure 10.

### 4.3. Experimental Results in the Vertical Test Section

**Figure 11:**
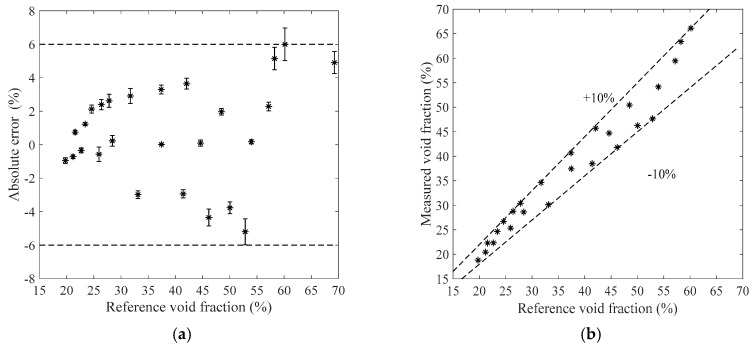
The measurement results of the bubble flow in the vertical test section when the liquid mass flowrate is 500 kg/h. (**a**) Absolute errors of the void fraction; (**b**) Comparison between the measured and reference void fractions.

**Figure 12:**
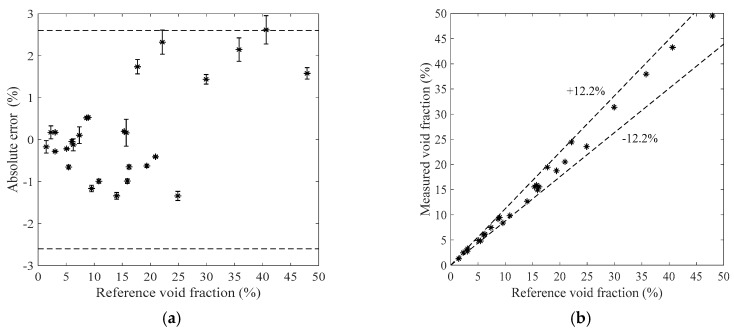
The measurement results of the bubble flow in the vertical test section when the liquid mass flowrate is 900 kg/h. (**a**) Absolute errors of the void fraction; (**b**) Comparison between the measured and reference void fractions.

**Figure 13:**
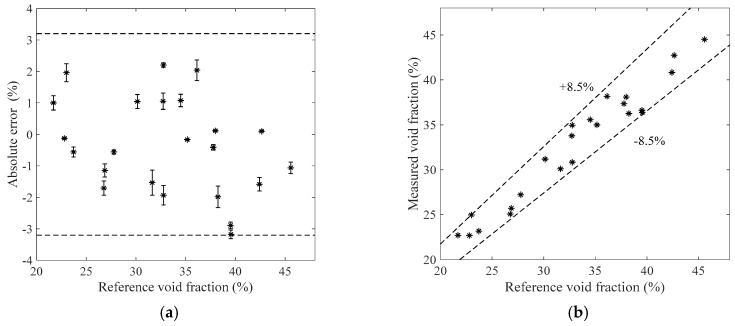
The measurement results of the bubble flow in the vertical test section when the liquid mass flowrate is 1100 kg/h. (**a**) Absolute errors of the void fraction; (**b**) Comparison between the measured and reference void fractions.

**Figure 14:**
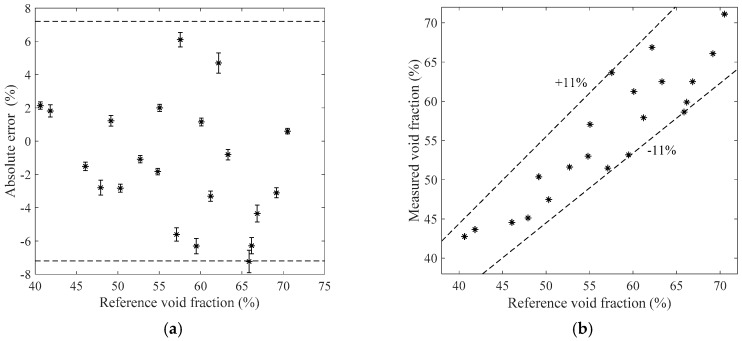
The measurement results of the bubble flow in the vertical test section when the liquid mass flowrate is 1300 kg/h. (**a**) Absolute errors of the void fraction; (**b**) Comparison between the measured and reference void fractions.

Figure 11, Figure 12, Figure 13 and Figure 14 show the measurement results of the void fraction measurement in the vertical test section with various void fraction ranges. The absolute errors of the bubble flow in the vertical test section are depicted in Figure 11a, Figure 12a, Figure 13a and Figure 14a. The relative errors are also shown in Figure 11b, Figure 12b, Figure 13b and Figure 14b. The maximum absolute error is 7.2% while the maximum relative error is ±12.2%.

## 5. Discussion

The experimental results have shown that the stratified flow can be observed in the horizontal test section when the liquid mass flowrate is under 900 kg/h. The bubble flows can all be observed in the horizontal and vertical test sections. Compared with the measurement results of the stratified flow and bubble flow in the horizontal section, the relative error of the stratified flow is less than that of the bubble flow. This is mainly because of the influence of the refraction of lights in the bubble flow. In the horizontal test section, the flow pattern of the two-phase CO_2_ flow is stratified flow when the mass flowrate of the liquid is less than 900 kg/h. The interface between the gas and liquid is distinct, which has little effect on the laser beams. When the mass flowrate of the liquid is larger than 900 kg/h, the continuous CO_2_ gas becomes bubbles due to the high pressure in the pipe, thereby leading to the formation of a bubble flow. The interface between the bubbles and the liquid in the bubble flow has a large effect on the laser beams. Moreover, the relative errors of the measurement results of the bubble flow in horizontal test section with the liquid mass flowrate of 1300 kg/h are larger than that with the liquid mass flow rate of 1100 kg/h. This is because the size of the bubbles decreases with the increase of the liquid mass flowrate, which causes a greater effect on the laser beam. In the vertical test section, the flow pattern is mainly bubble flow. Due to the influence of the slip ratio and the interface of the bubbles, the measurement method performs slightly less well in the vertical section.

In this study, an infrared laser and a photodiode array sensor are used to obtain the gas void fraction of the gas-liquid two-phase CO_2_ flow in pipelines. However, in industrial applications, impurities in CO_2_ produced from using different capture methods may lead to changes in the phase properties of the CO_2_ flow. In addition, the particular infrared ray absorption bands of impurities are different. The relationships between the infrared attenuation and the void fraction of the CO_2_ flow with various impurities should be considered. Therefore, the void fraction measurement under the conditions with impurities will be conducted in future research. Meanwhile, the applications of the proposed optical system on the measurement of other gas-liquid two-phase flows still have many limits, such as the diameter of pipelines, the wavelength of laser beams and the transmission medium. Previous studies [27,28] have demonstrated that gas-liquid two-phase flows in large-diameter pipes show different fluid structure behaviors compared with small pipes. A more comprehensive investigation will be carried out to further evaluate the proposed technique.

## 6. Conclusions

In this paper, the investigations of the gas void fraction measurement of the gas-liquid two-phase CO_2_ flow have been carried out using an infrared laser coupled with a photodiode array sensor. The photodiode array is designed to be parallel in two layers, but they cross each other along the pipe axis to avoid the dead space of the laser beams. The experiments were undertaken on a CO_2_ two-phase flow test rig. The liquid mass flowrate is set to 500 kg/h, 900 kg/h, 1100 kg/h and 1300 kg/h respectively, resulting in a gas void fraction ranging from 0% to 69%. Both the stratified flow and bubble flow are investigated on the horizontal test section, while the bubble flow is only investigated on the vertical test section. The relative errors of the stratified flow and the bubble flow are within ±12.2% and ±10.6%, respectively. The relative errors of the bubble flow in the vertical test section are under ±12.2%.

The measurement results have demonstrated that the developed measurement system is reliable and effective in measuring the gas void fraction in the two-phase CO_2_ flow. The measurement system has the advantages of having a low cost and a structural simplicity, and thus it can be a potential replacement for the expensive optical measurement system. Moreover, the proposed method can also be a useful reference for other applications of two-phase flow measurement, such as for oil-water two-phase flow and air-water two-phase flow.
